# Case Managers' Experiences of Engagement With Service Users in UK Early Intervention Services for First‐Episode Psychosis: A Hermeneutic Interpretive Phenomenological Analysis Study

**DOI:** 10.1111/eip.70098

**Published:** 2025-10-02

**Authors:** Paul Henderson, Suzanne Hodge, Bill Sellwood

**Affiliations:** ^1^ Faculty of Health, Social Care, and Medicine Edge Hill University Ormskirk UK; ^2^ Division of Health Research Lancaster University Lancaster UK

**Keywords:** case managers, early intervention services, engagement, experiences, first‐ episode psychosis, IPA, service users

## Abstract

**Aim:**

Staff engagement with individuals with first‐episode psychosis (FEP) in early intervention services (EIS) settings is important to reduce the longer‐term impact of this condition and promote recovery. There is a paucity of qualitative evidence exploring engagement from the perspectives of staff in these settings. This study aimed to explore and understand Case Managers' experiences of engagement with service users in EIS settings in the United Kingdom.

**Method:**

Hermeneutic interpretive phenomenological analysis (IPA) using semi‐structured interviews with purposively sampled EIS case managers with mental health nursing backgrounds from a single NHS Trust in the North of England. Data analysis followed IPA's six stages.

**Results:**

Five master themes were identified as being key to effective engagement: (1) Being on the same page, (2) Engagement as an interpersonal relationship, (3) Managing self and emotions, (4) The practicalities of engagement and (5) The impact of organisational factors upon engagement. Engagement was experienced as multi‐faceted, complex and changeable. Key engagement strategies identified as helpful were trust, therapeutic dialogue, shared understanding, collaboration and practical approaches.

**Conclusion:**

Engagement in the context of EIS can be defined as a mutually beneficial connection that takes place between service users and staff which allows for the implementation of treatment. Engagement strategies are an important consideration to reduce the potentially devastating impact of FEP, and to facilitate recovery. Further exploratory research should be conducted across multiple settings to further build on and understand engagement within the wider EIS context.

## 1 Introduction

First‐episode psychosis (FEP) typically begins in a person's mid to late teens and early twenties and can be characterised by symptoms that include hallucinations, paranoia, delusional ideas, disorganised speech or social withdrawal (Bromley et al. 2015; Chandra et al. 2018; England 2023). To address the impact of FEP, specialist mental health teams such as early intervention services (EIS) were set up in the United Kingdom during the 1990s to provide intensive support and treatment for individuals experiencing a first episode (Birchwood et al. 2002; French et al. 2010; Tindall et al. 2020).

A key principle of EIS is that treatment should begin as soon as possible during the course of FEP to prevent the development of severe symptomatic and functional impairments known to occur in major mental illnesses such as schizophrenia (McGorry 2000; McGorry et al. 2008; National Institute for Clinical Excellence (NICE) 2024). Thus, engagement of individuals with FEP with early interventions and treatment is fundamental to reduce the devastating, longer term impact of this condition upon individuals and their families, and facilitate a more favourable recovery (Birchwood et al. 2002). However, despite the efforts of mental health professionals to engage with individuals within this critical early treatment period, some individuals may either only partially engage or disengage due to factors such as poor insight, substance misuse, fear or stigma (Lecomte et al. 2008; Tindall et al. 2019). Up to 50% of individuals who use any mental health service disengage, being particularly problematic with young adults (Lal and Malla 2015; Mascayano et al. 2021). Disengagement by young adults may arise from difficulties coming to terms with a psychiatric diagnosis due to the associated stigma and their questioning the usefulness of professional support (O'Brien et al. 2009; Gulliver et al. 2010; Kular et al. 2019). Up to 60% of individuals diagnosed with FEP will disengage with EISs throughout their three‐year period of care (Tait et al. 2003; Tindall et al. 2015, 2020). Engagement with individuals who are experiencing FEP is particularly challenging for mental health professionals perhaps more so than with any other mental health disorder (Birchwood et al. 2002; Kim et al. 2019).

A range of attitudes, behaviours and skills have been identified as optimal for mental health professionals to successfully engage with individuals who use EISs. For example, developing a positive relationship based on trust, rapport and respect has been identified as a key factor influencing and enhancing engagement relationships between service providers and users and has implications for successful treatment outcomes (Tindall et al. 2019). Furthermore, individuals successfully engage with mental health professionals in EIS settings when they feel valued, listened to and understood (Stewart 2012; Huurman et al. 2023).

Given that engagement is not solely the responsibility of service users and draws attention to the practitioner's active role in this process, there is limited literature that has explored case managers' views and experiences of engagement with service users within EIS settings internationally (Harris et al. 2011; Tindall et al. 2015, 2018, 2019, 2020). Case managers typically have the most contact with service users throughout their three‐year period of care (Stewart 2012; Tindall et al. 2015, 2018). Case managers working within EIS settings are well placed to provide valuable insights into why engagement may take place or not. Given the paucity of evidence around this issue, it is important to understand engagement from the perspectives of staff working in these services. More specifically, there are no known studies that have focused on the engagement experiences of case managers from mental health nursing backgrounds in EIS settings. Appreciating case managers with core professional qualifications in mental health nursing may further increase understanding of engagement and to draw useful comparisons to the engagement experiences of other professional groups such as occupational therapy, social work or clinical psychology (Tindall et al. 2019). Therefore, the key focus of this study was to gain greater understanding and insight of a case manager from a mental health nursing background and their experiences of engagement with service users within EIS settings, and how they experience the process of engagement and the various approaches that they use to establish contact and engagement.

## 2 Materials and Methods

### 2.1 Study Design

A qualitative study underpinned by a hermeneutic interpretive phenomenological analysis (IPA) approach using audio‐recorded semi‐structured interviews was undertaken (Smith et al. 2009). IPA was selected due to its focus upon the interpretation, understanding and uniqueness of lived experience (Smith et al. 2009). The exploratory qualities of IPA are said to provide the potential for greater insight into topics of which little is known or understood (Eatough and Smith 2006; Smith et al. 2009; Tompkins and Eatough 2012). A further distinctive feature of IPA is based upon the premise to understand not only the subjective meanings held by individuals but also to account for those of the researchers (Pietkiewicz and Smith 2012). IPA assumes that the researcher's own knowledge, experiences and perceptions of the topic in question should be taken into account, but not to the extent that they form the focus of the research. Hence, the two‐stage process of IPA, the ‘double hermeneutic’ involves the participant attempting to make sense of their experiences, as well as the researcher, attempting to make sense of their sense‐making processes (Smith et al. 2009).

It was also considered that the complexity of the issue under investigation (engagement) would not be amenable to a more generalised focus, as each case manager's experiences of engagement are uniquely situated, discrete and subjective due to the fluid, dynamic and changeable nature of engagement. Thus, by deciding upon IPA as the primary research method for this study, it was anticipated that this would provide deeper interpretation of personal accounts, leading to potential new insights and understandings. The epistemological stance was based on social constructionism, acknowledging that through their social dealings, individuals produce a range of possible social constructions of events to construct their own versions of reality (Crotty 1998; Burr 2015; Smith et al. 2009).

### 2.2 Sampling and Recruitment

Recruitment occurred via three EIS spoke teams operating within one NHS Trust in the North of England. Following a short presentation (by P.H., lead researcher) about the research, potential participants (case managers) were sent an email with a recruitment flyer and participant information sheet. Purposive sampling techniques were used to recruit case managers from mental health nursing backgrounds who had knowledge, experience and expertise in engagement with service users in EIS settings. Individuals were eligible to take part in the study if they met the following criteria: currently employed as a case manager within an EIS setting; had more than 1 year of clinical experience in an EIS setting; and had a professional qualification in mental health nursing.

### 2.3 Data Collection

Semi‐structured, one‐to‐one interviews (averaging 40 min) were undertaken by PH between November 2017 and February 2018. In line with the participants' wishes, all interviews took place in a private office space at their place of work during normal office hours. A topic guide designed in line with Smith et al.'s (2009) interpretative phenomenological analysis (IPA) recommendations consisted of 10 open questions along with possible prompts. Consistent with IPA guidance (Smith et al. 2009), a warm‐up question was used: ‘To start with, I would like to know a little more about you and how you came to work in early intervention services?*’*. Another core opening question was asked: ‘In your own words, can you tell me what the term “engagement” means to you in your role in early intervention?*’* (Table 1). Following these two opening questions, seven more specific questions and a closing question, each with prompts, were asked. All interviews were audio‐recorded, and the audio files were transferred to a password‐protected computer (Pietkiewicz and Smith 2012; Smith et al. 2009). Once all interviews had been completed, all the recordings were transcribed verbatim. All interviews were conducted by P.H. P.H. kept a reflexive account of data collection allowing for biases, assumptions and ideas to be documented and scrutinised during discussions (Table 2). Since researchers utilising IPA are always part of both data collection and interpretation of the results, P.H. openly discussed and documented any relevant preconceptions and knowledge relating to engagement experiences with other team members.

**TABLE 1:** Interview topic guide.

Opening questions To start with, I would like to know a little more about you and how you came to work in early intervention services In your own words, can you tell me what the term ‘engagement’ means to you in your role in early intervention? *Possible prompts*: Trust, collaboration, co‐operation, two‐way relationship Specific questions 3 What are the most important processes for engaging service users? *Possible prompts* **:** Trust? Being friendly? approachable? collaborative? flexible? creative? 4 Can you tell me what you do to engage with service users? *Possible prompts:* What works or helps? What are the opportunities? Building rapport? 5 How do you know if a service user is engaging well with you and the early intervention team? *Possible prompts:* What happens? In what way? Ask for some examples. Are measurement tools such as ‘Service user engagement Scale’ (SES) used to gauge level and frequency of contact? 6 What makes it hard to engage with service users? *Possible prompts:* What gets in the way? What do you do? What might make it worse? 7 How do you know if your interaction with a service user is positive? *Possible prompts:* In what ways? How does it compare to a difficult interaction? 8 Now I'm going to ask you about attendance of arranged meetings or visits. Have there been any instances where a service user did not attend an appointment with you but wanted to? *Possible prompts*: What are the reasons? What do you do? 9 Have there been any instances when a service user intentionally chose not to attend an appointment with you? *Possible prompts*: What happened? What were the challenges? What did you do? Closing question 10 Is there anything I haven't asked that you think is important when thinking about how case managers engage people with early intervention services? Follow‐up question 11 Great thank you for that—is there anything else you would like to add before we finish the interview?

**TABLE 2:** Example of a reflexive diary entry.

*I am a former employee of the host NHS Trust having worked as a case manager in an EIS setting. I am a registered nurse (mental health) and my own practice history largely relates to the care and treatment of young people with first‐episode psychosis. As I develop my research study and methods, I am conscious of the need to critically reflect upon my role as a researcher and the impact that this has on participants especially those who have been known to me in my previous role. I am aware that my previous role as an EIS case manager may have an impact on recruitment strategies‐ will some staff be comfortable taking part since some will have worked with me previously? During interviews, I will need to reflexively recognise my influence as a former EIS case manager and respond to such issues as interactions take place. Additionally, I will need to fully acknowledge and respond to my prior and unfounded assumptions and pre‐conceptions with the analysis of the interview data*.

*Note:* The italics value signifies the use of the primary author's (PH) authentic voice in the process of reflexivity.

### 2.4 Ethical Considerations

Ethics approval was obtained from P.H.'s educational institution and research governance approval from the Health Research Authority. To ensure informed consent, prospective participants were provided with verbal and written information about the research. Consent was also revisited prior to interviews taking place, with written consent being sought at the point of interviewing (Smith et al. 2009). Participants had the right to withdraw from the study, up to the point of data being analysed.

### 2.5 Data Analysis

The IPA method outlined by Smith and Osborn (2008) was utilised to guide analysis, following six key stages. In keeping with IPA's idiographic principles, the sample size of this study (*n* = 7) was small enough to allow for detailed case‐by‐case analysis of individual accounts (Miller et al. 2018). The aim of IPA is not to generalise about larger populations, but to arrive at more general claims cautiously, and only after analysis of individual cases based on relatively small samples (Smith and Osborn 2008; Smith et al. 2009). IPA uses an idiographic approach in that the meanings that each individual attaches to their experiences are explored (Smith and Osborn 2008). During this stage of IPA, researchers engage in a double hermeneutic, trying to make sense of the participants trying to make sense of their own experiences (Smith and Osborn 2008). IPA studies pay close attention to patterns in participants' experiences, considering the ways in which meaning is made of those experiences, and interpreting such experiences within social and theoretical contexts (Larkin and Thompson 2012). P.H. lead the analysis with scrutiny from other team members that included open and frank discussions around transparency and trustworthiness of this process. Regular reflective discussions around analytical choices and re‐examination of transcripts aimed to ensure that themes emerging from the analysis reflected the participants' individual accounts.

For Stage 1, each interview transcript was read and re‐read several times to allow for immersion and familiarity with the participant's account. In Stage 2, the left‐hand margin of each transcript was used to make initial notes, comments and underline text in the main body on anything relevant to the research focus, i.e., participants' perceptions of and experiences of engagement. For each piece of underlined text, an accompanying summarising note in the left‐hand margin of the transcript was made to capture its relevance for the participant, staying as close as possible to the participant's own meanings. In doing this, care was taken to avoid making personal interpretations or value‐based judgements. Initial notes included commentary on issues that all participants discussed as important to their engagement experiences, such as therapeutic relationships, work‐related schedules, professional values, personal feelings and significant events. In Stage 3, the right‐hand margin of each transcript was used to reduce the initial annotations into succinct summary statements that captured how the participant made sense of their experience.

In Stage 4, patterns and connections were sought across transcripts to establish similarities, differences and groupings. Identified themes that arose, such as ‘being person‐centred’, ‘being genuine’ and ‘listening’ gravitated more naturally together to begin developing a super‐ordinate theme such as ‘therapeutic dialogue’. Stage 5 involved moving to the next transcript and starting the whole process afresh by repeating stages one to four. Each transcript was re‐visited several times to look at logical or appropriate connections between its emergent themes that would clearly convey sense and meaning of the complex and unpredictable nature of engagement as experienced by each participant. Stage 6 involved looking for patterns, similarities and differences to develop master themes that were applicable across the whole group, mapping the super‐ordinate themes together across the transcripts (Smith et al. 2009). Super‐ordinate themes such as ‘laying the foundations’ and ‘co‐creating engagement’ were identified with colour highlighting to show connections across more than one case. Anonymised quotations are used to illustrate themes, and each participant is coded as case manager (CM).

## 3 Results

Seven participants, age range 29–63 years, from across three EIS teams with 11 years (mean = 7 years) of practice experience within the service participated (Table 3). Five master themes were generated: (1) Being on the same page, (2) engagement as an interpersonal relationship, (3) managing self and emotions, (4) the practicalities of engagement and (5) the impact of organisational factors upon engagement.

**TABLE 3:** Participant characteristics.

Participant	Age range	Gender	Ethnicity	Profession	Length of experience in EIS
1	21–35	Female	White British	Registered mental health nurse	8 years
2	50–64	Female	White British	Registered mental health nurse	8 years
3	35–49	Female	White British	Registered mental health nurse	4 years
4	50–64	Female	White British	Registered mental health nurse	8 years
5	35–49	Male	White British	Registered mental health nurse	8 years
6	35–49	Female	White British	Registered mental health nurse	11 years
7	35–49	Female	White British	Registered mental health nurse	1.5 years

### 3.1 ‘Being on the same page’

All case managers asserted that for engagement to be successful, there was a need to develop shared understanding and respect for each other's contribution. CM1 emphasised that service users should show the same level of commitment and responsibility for arranging appointments as she did, ‘if I can't attend, I'll let you know’, *‘I'd also like it if you could do the same’*.CM3 also described the importance of listening to and understanding the service user's wishes as some prefer ‘minimal’ future contact:

….*they're happy for us to maybe go round every few weeks and just check in with them, without focusing in any detail on recovery work*

Other case managers identified that being clear and upfront from the outset about the purpose of their role was crucial to set the scene and develop shared understanding about collaboration:

……*I will not tell you something just to please you, we are here to work together and I will be honest with you, it might be uncomfortable but we will work through that honesty, that uncomfortableness together, and I take it from there*. (CM2)

However, in term of new insights, some case managers also expressed concern and caution in terms of the impact and influence of their age and generational belief systems when working with the broad age range of service users on EIS caseloads, particularly younger people. As six out of seven of the case managers who took part in this study were aged between 35 and 63 years, it was highlighted how the age of the practitioner and service user could potentially be a hindering factor in relation to being ‘on the same page’ in relation to engagement.

### 3.2 ‘Engagement as an interpersonal relationship’

All case managers recognised that they needed to build genuine person‐centred connections with service users such as being ‘there for the right reasons and…those basic things…being genuine, warm and listen, listening is a big thing*’* (CM5). Genuine human connection was consistently identified as key to successful engagement.

…*I try and be honest and genuine, you know things like if you need to go out and have a cigarette or that sort of thing, it can be quite helpful can't it to, or if you need a break at any time or if you know, that you'd like to call a halt to it or sort of thing… just trying to create a relaxed atmosphere, being open and honest*. (CM4)

Conversely, CM6 noted that some service users with all sorts of trust issues often ‘tested’ the sincerity and loyalty of case managers as a result of their previous experiences they perceived as having been negative as they ‘actually want to see you work for it sometimes, because they've been let down so many times before*’*. However, although case managers believed in being person‐centred to develop trusting relationships, they were not necessarily family‐centred. They perceived the presence and involvement of some family members to be less helpful, somewhat intrusive, and not always beneficial to the building of trusting relationships with service users as this could create potential barriers to further opportunities for engagement. Case managers proposed that some service users may not be ready to openly discuss their problems in the presence of their family members, suggesting that this may impede some individuals from readily engaging and building trust with their case manager. This was noted as being a major challenge as case managers were mindful of the importance of being able to build positive working relationships with families and caregivers to develop a greater level of knowledge about mental health, support strategies and treatment interventions.

### 3.3 ‘Managing self and emotions’

All case managers described experiencing high levels of emotional impact in connecting with service users and how this could affect their ability to engage. CM4 described how engagement can be hard for some service users such as those with:

….*difficult personalities or personality type disorder things, and I do think that does make engagement hard in some respects, or it's the internal frustration you feel*. (CM4)

Another CM explained that individuals with a forensic history could be challenging to engage as:

….*the crimes might be….violent crimes, so you're going into somebody's area knowing that there's a potential that they could be quite unwell and that they've got this past history, so that, I think that would play for me*. (CM7)

Intensively working alongside service users over long periods of time led to exit grief for some case managers:

*I suppose at the end of three years there is some aspect really of almost a grief really isn't there? Because you are saying goodbye to somebody that you've worked intensely with, or you should have worked intensely with over three years*. (CM2)

Case managers identified an emotional cost of working closely alongside the service user during their three‐year period of care with EIS. Words used such as *‘*grief’, ‘saying goodbye’ and *‘*letting go’ all convey a sense of sadness and loss. However, case managers generally recognised that experiencing feelings such as sadness and loss were a predictable part of the job, due to investing much time and an intensive level of effort and support when working alongside service users and their families. Case managers also believed that as they invested much of themselves in their engagement work with service users, their experiences of working alongside some individuals with personality disorders could be seen as unrewarding and demoralising due to challenges such as rejection of support.

### 3.4 ‘The practicalities of engagement’

Engagement needed to be practically and materially useful to service users to meet their often‐changing needs, these included ‘…benefits, housing, all those practical needs as working on those does improve engagement if you work on those first’ (CM6). The need for flexibility was identified as being core to optimising engagement:

…*trying to be flexible with appointment times….we work eight till eight…and other people like service users may be busy or occupied in different hours*. (CM3)

Ensuring geographically convenient engagement was deemed important:

….*is it closer to home? Is it kind of, more convenient for them that they don't have to get on a bus for half an hour to an appointment or ask for a lift off somebody? So, seeing them in, in their own home can sometimes be, be more beneficial*. (CM1)

Case managers felt that there was no‐one size fits all, and that conventional timeframes such as nine to five office hours may not work for some service users on their caseloads due to other pressing commitments in their lives. They also stated that approaches needed to be open, bespoke, flexible and negotiable to optimise engagement rather than adhering to the requirements prescriptively set out by EIS policy protocols.

### 3.5 ‘The impact of organisational factors upon engagement’

Case managers candidly spoke about the influence and impact of EIS organisational factors upon the engagement process.

….*it's all about meeting targets….that takes away a lot of the job satisfaction because you're not able to treat people or spend the time with them that you'd like, and things are getting rushed*. (CM4)

Resource constraints also impacted frequency of engagement, although services such as assertive outreach teams supported daily contact:

….*we don't have the resources to do that (daily contact) so I think yeah, I had to step back and think you can't be there as much*. (CM6)

The changing ethos of EIS provision reported to be impacting engagement as EIS is: ‘…becoming more like….a mini‐crisis team and that is not the philosophy of EIS’ (CM2).

Case managers perceived that they were unable to fully perform the EIS case manager role that they had originally signed up for. The use of words such as ‘a mini‐crisis team*’* and ‘spoiling what is a very good service*’* highlights a sense of disappointment and frustration with senior managers whom they perceived as lacking understanding about the true nature and purpose of what EIS care should look like. The above extracts further draw attention to how case managers perceived that EIS was being forced to adopt an increasing level of crisis intervention as an approach due to reduced service resources, which further impacted upon their available time to offer therapeutically effective contact with service users. The importance of having more available time as a commodity to invest in the service user consistently emerged throughout this theme. The finite nature of a case manager's case work time within EIS settings due to service level factors was perceived to impact upon their capacity to engage and contributed to the overall quality and meaningfulness of their connection with service users.

## 4 Discussion

This study contributes a new, deeper understanding of the unique engagement experiences of case managers from mental health nursing backgrounds with service users in EIS settings in the United Kingdom. Using IPA allowed for deep interpretation of personal accounts, leading to new insights and understandings. All case managers experienced engagement as involving numerous, changeable processes that they endeavoured to carefully facilitate. Case managers proposed that effective interventions could only take place when genuine, trusting and collaborative relationships between service users and themselves were established. Although all case managers taking part in this study were experienced professionals, they also identified that reflection was key to building good engagement through a process of self‐awareness and being able to understand how their skills and abilities influenced and enabled successful engagement. Additionally, case managers attached importance to emotionally regulating themselves using strategies such as being patient, resourceful and proactive to cope and maintain resilience in the face of poor, episodic or non‐engagement issues.

The emotional costs described by case managers were highlighted as having the potential to impact upon the perceived quality of their engagement contact. The Tidal Model acknowledges the emotional challenges faced by some mental health practitioners and re‐iterates the importance of practitioners accessing debriefing to re‐evaluate their human qualities rather than their skills to effectively engage with individuals (Barker and Buchanan‐Barker 2005, 2010). This is an ongoing consideration for community mental health practices. It could be argued that this finding further reinforces the importance of utilising self‐protective, reflective and boundary management approaches to lessen the emotional impact resulting from the intensive levels of engagement work in EIS settings (Grant and Kinman 2014).

Case managers valued the importance of establishing a shared understanding to help service users make sense of their experiences. This concept not only included having the case manager endeavouring to understand the service user's unique experiences, but also being able to develop trust, rapport and genuine human connection. The findings resonate with the wider literature that emphasises the importance of the therapeutic alliance and person‐centred approaches such as empathy, unconditional positive regard, genuineness and viewing the service user as a person rather than an illness (Rogers 1980; Frank and Gunderson 1990; Horvath and Greenberg 1994). Case managers also described the importance of using less formal, flexible, practical and materially useful approaches to optimise engagement. This aligns with the proposal by Stroud and Parsons (2013) that rather than further developing specialist skills and knowledge when working alongside people with early psychosis, the service context and culture need to allow staff to engage in a more fluid, flexible and creative way to meet the complex, uncertain and changeable needs of this client group.

Organisational factors impacted how case managers perceived the value of engagement, leading to some apparently adopting more target‐driven and tick‐box orientated approaches rather than bespoke person‐centred care. This is perhaps unsurprising considering the operational difficulties reported elsewhere, particularly in delivering key EIS criteria (Dodgson and McGowan 2010). Operational factors include time, less complete and diverse skill mixes, fewer support and recovery workers and less dedicated medical staffing time (Dodgson and McGowan 2010). Arguably, EISs are more complex services to set up and implement than generic community mental health teams due to variations in local and regional needs (French et al. 2010). However, adapting EIS models to reflect and meet local needs can risk the intended core principles of EIS drifting away from the ethos of the service and its evidence base (National Institute for Mental Health in England 2003). Nonetheless, it is evident that some challenges are related to the commissioning and setting up of EISs rather than problems with how case managers deliver such care (Dodgson and McGowan 2010).

In view of the impact of organisational objectives, case managers would benefit from being able to dedicate more time for service user contact to further build and maintain positive engagement opportunities. However, while there may be service‐oriented challenges, these issues are neither unique to EISs, nor are they restricted to community mental health settings in general. Notwithstanding that, engagement with individuals with FEP is fundamental to achieving service delivery aims and facilitating recovery; EIS policies should revisit and review time allocation for case manager contact to build on effective engagement. Despite the study's strengths, findings are limited by a small number of participants and the fact that data was collected 7 years ago, albeit that the context of practice in which case managers are working is broadly unchanged. A further limitation of this study is that it was conducted in a single EIS setting in one NHS Trust. Despite the service's distribution over three large geographical areas and three separate EIS teams covering a large, diverse population, the findings represent an interpretation of case manager experiences in that setting. Additionally, all aspects of the data analysis were performed by a single researcher (P.H.) who was also a former, experienced case manager in an EIS setting. However, these processes were critically discussed and corroborated within research supervision.

## 5 Conclusion

Engagement in the context of EIS can be defined as a mutually beneficial connection that takes place between service users and staff, which allows for the implementation of treatment. Engagement strategies are an important means of reducing the potentially devastating impact of FEP and facilitating recovery. The findings suggest that case managers hold a common goal to develop genuine, person‐centred relationships with service users to meaningfully engage and achieve a sense of satisfaction and fulfilment in their professional roles. However, due to the intensive level of support provided by case managers over a 3‐year period in EIS settings, there is also a need to address the emotional impacts and implementation of supportive strategies to mitigate this. Further exploratory research should be conducted across multiple EIS settings to further build on and understand engagement within the wider context.

## Conflicts of Interest

The authors declare no conflicts of interest.

## Data Availability

The data that support the findings of this study are available from the corresponding author upon reasonable request.
